# Cationic Polymers with Tailored Structures for Rendering Polysaccharide-Based Materials Antimicrobial: An Overview

**DOI:** 10.3390/polym11081283

**Published:** 2019-08-01

**Authors:** Yuanfeng Pan, Qiuyang Xia, Huining Xiao

**Affiliations:** 1 Guangxi Key Laboratory of Petrochemical Resource Processing and Process Intensification Technology, School of Chemistry and Chemical Engineering, Guangxi University, Nanning 530004, China; 2 Department of Chemical Engineering, University of New Brunswick, Fredericton, NB E3B 5A3, Canada

**Keywords:** polysaccharide, antimicrobial, guanidine-based polymer, gemini, antimold

## Abstract

Antimicrobial polymers have attracted substantial interest due to high demands on improving the health of human beings via reducing the infection caused by various bacteria. The review presented herein focuses on rendering polysaccharides, mainly cellulosic-based materials and starch to some extent, antimicrobial via incorporating cationic polymers, guanidine-based types in particular. Extensive review on synthetic antimicrobial materials or plastic/textile has been given in the past. However, few review reports have been presented on antimicrobial polysaccharide, cellulosic-based materials, or paper packaging, especially. The current review fills the gap between synthetic materials and natural polysaccharides (cellulose, starch, and cyclodextrin) as substrates or functional additives for different applications. Among various antimicrobial polymers, particular attention in this review is paid to guanidine-based polymers and their derivatives, including copolymers, star polymer, and nanoparticles with core-shell structures. The review has also been extended to gemini surfactants and polymers. Cationic polymers with tailored structures can be incorporated into various products via surface grafting, wet-end addition, blending, or reactive extrusion, effectively addressing the dilemma of improving substrate properties and bacterial growth. Moreover, the pre-commercial trial conducted successfully for making antimicrobial paper packaging has also been addressed.

## 1. Introduction

**Figure 1:**
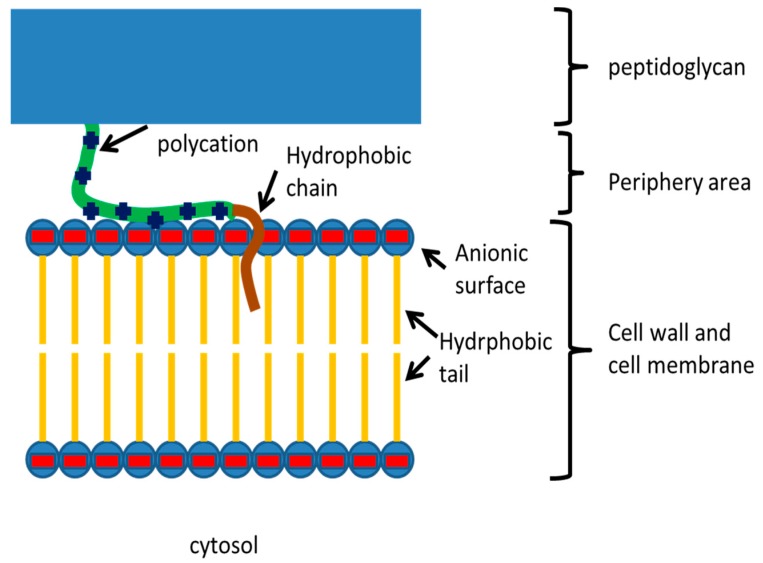
Proposed mechanism of bactericidal activity of polycation-hydrocarbon block copolymers. Reprinted with permission from ref. [2]. Copyright 2018, American Chemical Society.

Bacterial infection is a significant threat to human health today. Fighting against harmful bacteria is a great challenge, due to the development of resistance to antibiotics. Cationic polymers or polycations with the proper combination of cationic and hydrophobic chains are promising antimicrobial compounds, owing to their broad antibacterial spectrum and permanent antimicrobial activity [1,2]. To the best of our knowledge, although polycations have different antimicrobial efficiencies against different bacteria strains, no developed resistance has been reported. Cationic polymers can adhere to negatively charged surfaces of bacteria. This interaction is relatively selective, because mammalian cell surfaces are less negatively charged. Polycationic chains can be combined with other functional components that can kill and inhibit bacteria growth in different ways. The mechanism of killing was suggested to be adsorption of a positively charged layer onto a negatively charged cell membrane surface through electrostatic interaction, followed by weakening of the cell membrane by diffusion of a lipophilic alkyl chain [3,4,5,6]. This leads to loss of cytoplasmic contents and death of the cell (Figure 1).

Some excellent reviews on cationic polymers have been presented in the past, including the one from Kenawy et al. [7], though the application has not been specifically targeted to polysaccharide or cellulose-based materials. Klibanov et al. gave a concise review on polycations containing hydrophobic block coatings on material surfaces for antimicrobial purposes [8]. It was concluded that combining cationic and hydrophobic features in a polymer enhanced the stability of hydrophobic chains against massive aggregation. Rather, complex phase behaviors were observed in the past for ionic amphiphilic polymers [9]. Klibanov et al. also suggested that the hydrophobic and cationic parts should occupy optimal portions in a polymer to maximize antimicrobial ability. The role of the hydrophobic part is to diffuse into the hydrophobic core of the cell membrane and break the structure. Many studies have shown that too short a hydrophobic chain is not as effective as chains with optimal lengths [10,11,12]. Only cationic polymers, and not anionic or zwitterions, exhibit antibacterial activity. Noticeably, hydrophobic polycations are also toxic to viruses, with even relaxed constraints on hydrophobic content, molecular weight, and zwitterions charges [8]. Kugler et al. showed that for a surface coated with hydrophobic polycations, a minimum charge density threshold should be met for effective growth inhibition [13].

Guided by this antimicrobial mechanism, various cationic polymers with tailored or well-defined structures have been developed over the past decades, although the detail of whether—or how—these polymers penetrate through the peptidoglycan layer is still unknown and remains a subject under debate. To date, antimicrobial polymers have been extensively employed in the plastic and textile industries, but much less attention has been paid to cellulose-based paper products until recently. In fact, the paper industry needs innovative materials that are compatible with paper and can add value to the users. Antimicrobial papers can be used in scenarios that need high hygiene standards, such as hospitals, food packaging, food handling, and even banknotes, which greatly improve the health protection of users. While most traditional antimicrobial materials are based on a slow release of antibiotics or other antimicrobial compounds, these materials have life-time constraints because the antimicrobial compounds will finally drain out or leach off. In contrast, the idea of contact killing is attractive due to its persistent nature. This idea has already been commercialized in silver-coated antibacterial surfaces. Copper and copper oxide compounds are also a hot area of research in recent years. The advantage of using polymeric antimicrobial materials is that the antimicrobial units or segments can be tightly integrated into a polymeric network or backbone, whether synthetic or natural, with minimal influence on the physical properties and visual appearance, as well as without leaching the compounds to the environment. The major positively charged groups used in polycations are amines, quaternary ammonium, and phosphonium salts. Quanternary ammonium salt (QAS) and phosphonium are more commonly used in synthesizing antimicrobial polymers, because they are chemically more inert than amine groups, which have been comprehensively reviewed previously [14]. However, less attention has been paid to developing antimicrobial cellulose or paper products incorporated with polycations. The review presented herein mainly focuses on guanidine-based polymers and their derivatives for rendering cellulose fibers or paper antimicrobial. The review has also been extended to antimicrobial gemini surfactants or polymers, as well as synthetic polymers (like polypropylene; PP) as substrates. Guanidine-based polymers possess some unique structures differing from conventional QAS or phosphonium-type polymers, with much higher antimicrobial efficiency and lower minimum inhibitory concentration (MIC). Moreover, guanidine-based polymers are highly water-soluble, and very applicable in fabricating cellulose-based or paper products in aqueous systems. The resulting paper can bear proper functional groups on the surface, providing flexibility in introducing additional functionalities for various applications—in particular, hygiene paper products.

## 2. Preparation of Guanidine and Biguanidine-Based Antimicrobial Polymers

Polymeric guanidines are one kind of polymer promising in inhibiting bacterial growth. The synthesis of a typical guanidine-based polymer, polyhexamethylene guanidine hydrochloride (PHGH), is accomplished by polycondensation of hexamethylenediamine chloride with dicyandiamide [15]. The molecular structure of the resulting polymer is often characterized using viscometry, nuclear magnetic resonance (NMR), and FT-IR. One monomer of PHGH contains a six carbon alkyl chain and a hydrochloride guanidine or biguanidine group. The number-average molecular weights of polymers are not high, ranging between 800 and 3500, depending on the duration of the reaction. Therefore, PHGH is sometimes referred to as an oligomer, instead of a polymer. Similarly, alkylated cationic oligomers with optimized antibacterial activity were also reported by Grace et al. [16].

**Figure 2:**
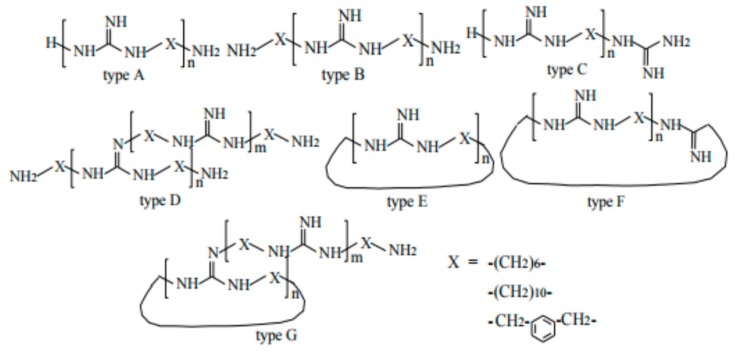
Seven possible structures of PHGH.

The seven possible structures of PHGH are illustrated in Figure 2. Overall, PHGH showed excellent antimicrobial activity in terms of minimal inhibition concentration (MIC) (as low as 8 ppm). Copolymerization was applied to modulate the properties of PHGH. The purpose was mainly to improve the physical properties of the final product. Wei et al. prepared guanidine containing acrylic fibers by copolymerizing guanidine oligomer and acrylonitrile, followed by wet-blend-spinning [17]. The resulting fiber showed better elasticity and better thermal stability, compared to pure acrylic fiber; 99% bacterial inhibition was achieved at 2.75 wt % of PHGH, possibly due to the distribution of PHGH among the interior and the surface of the fiber. The same copolymer can be readily applied to cellulose-based or paper products.

Li et al. prepared permanently antimicrobial polypropylene (PP–*g*–PHMG) by covalently bonding poly(hexamethylendiamine-guanidine hydrochloride) (PHMG) onto a polypropylene (PP) matrix using a melt grafting method in supercritical CO_2_ [18]. These authors further spun-bonded this material into non-woven fabrics and tested the antimicrobial activity. The antimicrobial activity of this fabric did not diminish in a 10-day water wash test. Animal tests showed no lethal or skin irritation effect. PHMG–PPGDE block copolymer was also synthesized via chemical reaction between PHMG and polypropylene glycol diglycidyl ether (PPGDE) [18]. The copolymer was amphiphilic and had excellent antibacterial activity. The as-prepared copolymer was incorporated into cellulose-based fibers (cotton) though physical absorption and chemical crosslinking. The resultant cotton fabric showed excellent antibacterial activity and washing resistance. In another report, Wei et al. [19] prepared antimicrobial polypropylene wax (PPW–*g*–PHMG) via melting reaction between polyhexamethylene guanidine(PHMG) and polypropylene wax-grafted maleic anhydride in a Hakke torque rheometer. The antibacterial activity against *Escherichia coli* was confirmed by a ring diffusion test. After being purified, the PP/PPW–*g*–PHMG sample became a non-leaching antimicrobial material with excellent and durable antimicrobial activity.

## 3. Surface Modification Using Guanidine and Biguanidine Polymers

**Figure 3:**
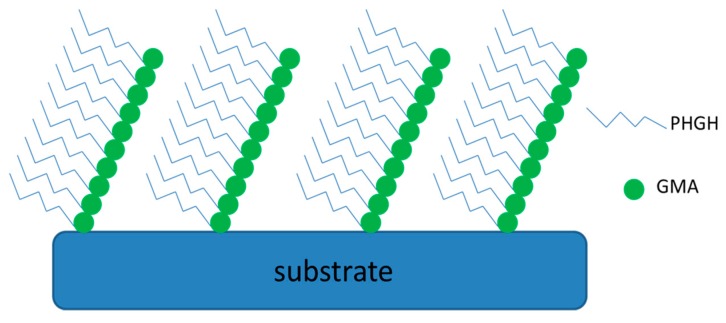
Schematic of attaching poly(GMA-PHGH) on a functionalized substrate.

PHGH was grafted on many different substrates, depending on specific applications. Guan et al., for the first time, grafted the guanidine-based polymer (PHGH) onto cellulose fibers via in situ polymerization [20]. Glycidyl methacrylate (GMA)-modified PHGH polymers were prepared as macromonomers for subsequent free-radical polymerization. GMA ends were initiated on the hydroxyl groups of cellulose fiber using ceric ammonium nitrate (CAN) as a free-radical initiator. The use of a Ce-type initiator ensures the grafting occurs mainly on the glucose units of cellulose, such as to minimize the homopolymerization of macromonomers in bulk phase. The resultant is a grafted comb-like structure on the cellulose fiber surface (see Figure 3). Atom force microscope (AFM) images revealed that grafted GMA–PHGH formed granules on the cellulose fiber surface. They found full inhibition of *E. coli* with 4% PHGH-grafted cellulose fibers, while 2-logs reduction could be achieved by 1% PHGH grafted on cellulose fibers. This work is a kind of pioneer work on rendering cellulose fibers antimicrobial using guanidine-based polymers—though work on treating synthetic fibers for textile using PHGH was already done previously. More interestingly, similar guanidine-based modification can be readily applied to starch, another type of polysaccharide abundant in nature. The resulting antimicrobial starch was successfully applied to rendering the packaging paper antimicrobial via a pre-commercial trial performed at FPInnvations Canada [21]. This was the first attempt to produce antimicrobial cellulose paper products using guanidine-modified starch as a functional wet-end additive in a pilot scale (up to 4.5 tons of antimicrobial packaging paper was produced within 4 h). The highly cationic-charged starch allowed itself to completely remain in fiber networks (i.e., nearly 100% retention). The resulting packaging paper showed superior antimicrobial activities, along with improved mechanical strengths.

As can be noticed from Figure 3, the density of polymer combs appears to be too high using GMA–PHGH macromonmer for synthesis. To decrease polymer density, a spacer needs to be introduced between neighboring PHGH chains. Wei et al. copolymerized polyhexamethylene guanidine hydrochloride (PHMG)–GMA macro-monomers with acrylonitrile as a spacer [22].

Wei et al. systematically studied the effects of GMA/PHMG ratio, solvent, temperature, and reaction time in reactions between PHMG and GMA on the GMA conversion rate. These parameters were optimal at 1.0, DMSO, 60 °C, and 60 h, respectively. ESI-TOF data showed that the products of PHMG/GMA reaction include three linear and four branched or cyclic species [22]. The (GMA–PHMG)–AN copolymer reduced cell content by more than 4 logs after contacting the suspension of Pseudomonas aeruginosa for 24 h. However, for water-soluble antimicrobial polymer, acrylamide (AM) is often used as a spacer to reduce the steric effect of PHGH combs.

**Figure 4:**
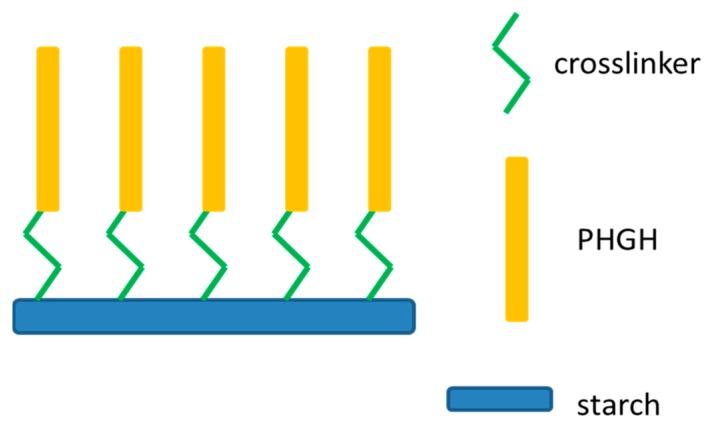
Grafting starch with multiple PHGH chains using a chemical crosslinker.

With different substrate and synthesis routes, Guan et al. [23] covalently linked whole PHGH chains onto gelatinized potato starch via coupling reaction through glycerol diglycidyl ether (GDE) (Figure 4). The optimum reaction time, temperature, pH, and starch/PHGH weight ratio for maximum conversion rate were identified. The paper treated with modified starch had superior bactericidal activity against *E. coli* ATCC11229 in a shaking flask test. They also characterized the interaction between cationic PHGH-starch and cellulose using adsorption isotherm and atomic force microscopy [24]. It was found that with an increase of charge density on starch, the adsorption capacity first increased, followed by a slow decrease. This was attributed to the different configuration of starch aggregate with different charge density. They also showed that the interaction between cationic starch and cellulose fibers was majorly electrostatic, which ensured the high retention of antimicrobial-starch on cellulose via the wet-end addition. Apart from the wet-end addition, the antimicrobial starch could be applied for the surface coating, including starch-based flexible coating for food packaging paper [25].

## 4. Antimicrobial Beads or Latex and Star Polymer Containing Guanidine Chains

**Figure 5:**
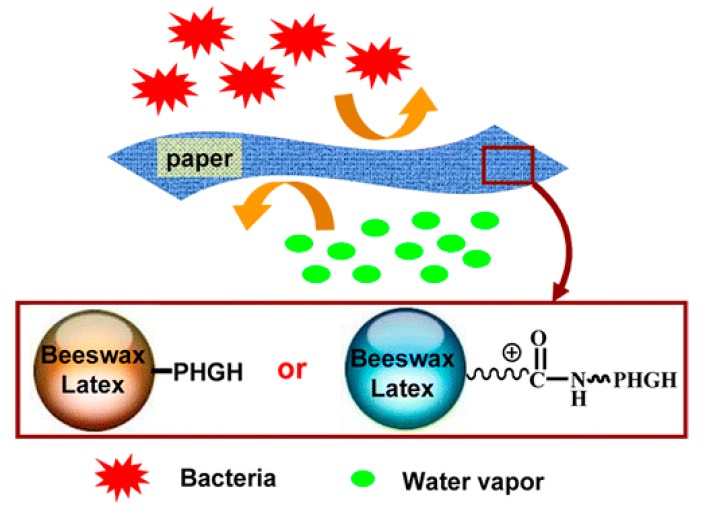
PHGH modified beeswax latex is incorporated into the paper sheet to increase the water resistance. Bacteria growth can be inhibited on the paper. Reprinted with permission from ref. [26]. Copyright 2013, American Chemical Society.

Fillers or pigments have been extensively used in papermaking and coated paper products. Adopting a similar idea, antimicrobial beads or latex particles have been developed in the past decade, specifically for rendering cellulose fibers or paper antimicrobial, either via wet-end addition or coating on paper surface. Zhang et al. [26] grafted PHGH on beeswax latex beads, with or without amphoteric surfactant. These kinds of beads endowed both water repelling and antimicrobial activity when incorporated into cellulose fiber networks or paper products. *N*-(3-dimethylaminopropyl)-*N*′-ethylcarbodiimide hydrochloride (EDC) was used as an effective crosslinker between beeswax and PHGH. Moreover, they created beeswax and carnauba wax latex beads, grafted with PHGH or polyhexanide, by coupling reaction using EDC as a coupling reagent [27]. These beads can effectively reduce the water vapor transmission rate of paper by one order of magnitude; while the bacterial inhibition test confirmed the excellent antibacterial activity of the beads, thus enhancing the multi-barrier properties of paper which is of great importance in food packaging (Figure 5). Xu et al. [28] prepared antimicrobial polyethylene wax (PEW) emulsions by emulsifying polyethylene wax grafted with PHGH (PEW–*g*–PHGH). The emulsion was used as a wet-end additive or coating material for fabricating hydrophobic and antimold hand-sheets or paper. The treated paper showed improved hydrophobicity and good antibacterial activity.

Pan et al. [29,30] created a latex bead with core-shell structures by copolymerizing butyl acrylate (BA) and ethylhexylacrylate (EHA), then adding a GMA–PHGH block over a seeded emulsion polymerization. Ethylene glycol dimethacrylate (EGDMA) was used as a crosslinker among monomers. The resultant was latex with a PBA–*co*–EHA core and charged GPHGH shell. The sizes of latex were between 60 to 160 nm, depending on amount of GPHGH.

The MIC of latex containing 30.2 wt % GPHGH can be as low as about 6.25 ppm, even better than pure PHGH. This is speculated to be due to nano-sized latex particles within the antimicrobial shell, which tends to maximize antimicrobial performance. These latexes can also be strongly adsorbed at cellulose fiber due to electrostatic interaction between the highly cationic shell of the latex and the anionic-charged fiber surface. The antimicrobial latex-loaded cellulose fiber also exhibited excellent antimicrobial activity, even when the dosage of latex on fiber was only 0.1 wt %. The key advantages of latex with a soft core (i.e., low glass transition temperature *T*_g_) is to facilitate the filming of the beads on fiber or paper surfaces, regardless of wet-end addition or coating.

**Figure 6:**
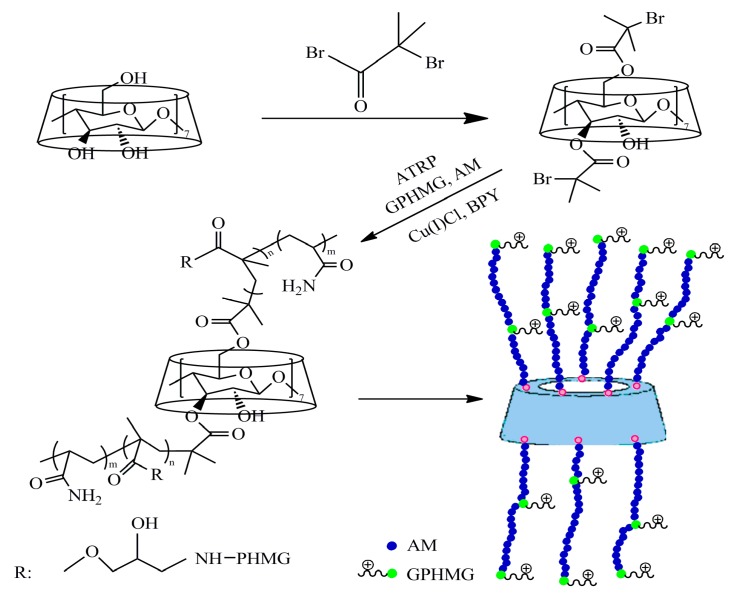
Antimicrobial/antiviral star polymer prepared via ATRP using Br-β-cyclodextrin as a macro-initiator. Adapted with permission from ref. [31]. Copyright 2019, John Wiley & Sons.

To further improve the antimicrobial activity, Pan et al. [31] synthesized a novel star polymer from cyclodextrin and PHGH–GMA macromonomer, via a living or atom transfer free-radical polymerization (ATRP). In this synthesis via living free-radicals, the cyclodextrin was a multifunctional macroinitiator, and acrylamide was the crosslinker between cyclodextrin and PHGH–GMA (Figure 6). The MIC of this star polymer against *E. coli* was as low as 0.78 ppm when charge density was high, which is higher than most guanidine-based polymers reported elsewhere. This ultra-high antimicrobial activity is attributed to a high number of PHGH in a local volume. This star polymer also showed improved antiviral activity. Pan et al. [31] found that star polymers with comparable amounts of PHGH possess excellent antimicrobial activity, which, however, strongly depends on the topological structure (i.e., the arm number and the monomer ratio) of the composing copolymers. The results show that the highest antimicrobial activity is achieved by the star-like copolymer with the monomer ratio of 20:3 (AM:PHGH, mol/mol), while the number of functional arms is fixed at eight. Similarly, Exley et al. [32] used eversible addition fragmentation chain transfer (RAFT) polymerization, another type of living free-radical polymerization, to prepare antimicrobial peptide mimicking primary amine and guanidine-containing copolymers. Moreover, a ring-opening metathesis polymerization (ROMP) was also utilized for preparing antimicrobial polymers, as reported by Lienkamp et al. [33].

**Figure 7:**
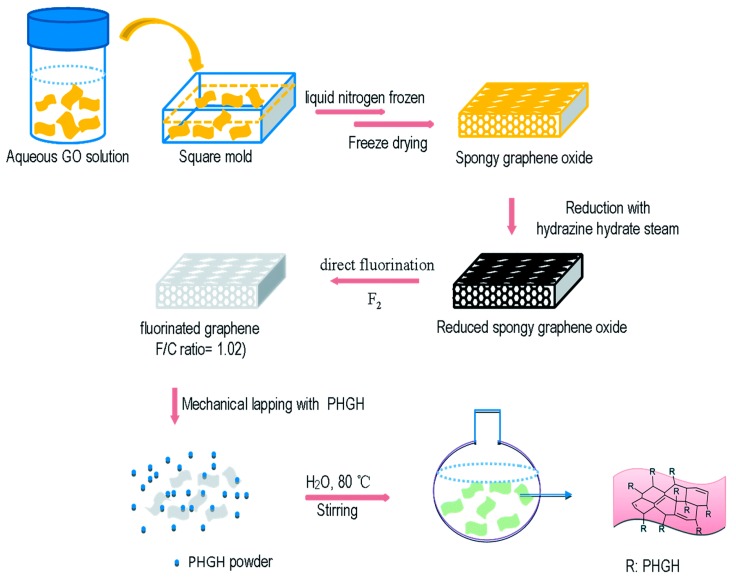
Grafting PHGH on fluorinated graphene surface. One thin dimension of fluorinated graphene could improve the membrane-breaking ability when combined with PHGH. Adapted with permission from ref. [34]. Copyright 2019, RSC Publishing.

Different from using polymer as a carrier for antimicrobial functional group, Wang et al. [34] prepared graphene oxide (GO), fluorinated graphene (FG), and guanidine-modified graphene (PHGH–G) (see Figure 7 for the process of modification), and examined their antimicrobial activities. 

It was found that the superior antibacterial activity of PHGH–G was achieved, even with low-density of PHGH on graphene when the graphene derivatives were dispersed in PBS during antimicrobial testing. AFM images show that PHGH–G can wrap onto the surface of bacteria and destroy the cell membrane. On the contrary, GO and FG can wrap onto the cell surface but cannot destroy the cell membrane. Based on similar guanidyl-functionalized graphene, Zhang et al. [35] also recently attempted to prepare an ultrafiltration membrane with superior permselective, antifouling, and antibacterial properties for water treatment. This is another example of antimicrobial-modified graphene for pathogen deactivation.

## 5. Complexation of PHGH with Other Polymers

Some physical interactions can be utilized to form PHGH-containing structures. The major interaction is electrostatic, while van der Waal′s force and hydrogen bonding could also exist. The preparation of such complexes is much faster and more convenient, with proper adjustment of physical environment.

Pan et al. [36] developed a thermal responsive layer-by-layer (LBL) assembly composed of ionic modified guanidine polymer (PHGH), poly-diallyldimethyl-ammoniumchloride (PDADMAC), and acetalyzed poly(vinyl alcohol)/sodium acrylate (APVA–*co*–AANa). The layers were both coated on silicon wafer and cellulose fiber. The antimicrobial activity of PHGH in LBL film with that of poly-diallyldimethyl-ammoniumchloride (PDADMAC) in LBL film was compared and contrasted. PHGH-containing film showed superior inhibition effects. Moreover, acetalyzed poly(vinyl alcohol) is a temperature responsive polymer [37]. The roughness of resulting antimicrobial LBL film is controllable under different temperatures.

Qian et al. produced a complex of EPHGH and carboxymethyl cellulose [38], utilizing electrostatic interaction. The complexation effectively improved the wet-strength of cellulose fiber networks. It was found that hexamethylene diamine (HMDA) content has a very significant effect on antimicrobial activity of the polymer and complex. The hand-sheet containing the antimicrobial polymer complex was also produced. The wet tensile strength of the hand-sheet was enhanced by five times, demonstrating the dual-functions of the complexes in enhancing both antimicrobial activity and wet-strength. This is mainly due to the similarity between HMDA and the diamine used for preparing the PAE type of wet-strength agent which has been well received in the alkaline papermaking process.

Similarly, Sun et al. [39] produced chitosan-guanidine polymer complex that showed a synergistic effect on antimicrobial activity. Both PHGH and crosslinked PHGH are used to form a complex with chitosan, using tripolyphosphate (TPP) as a crosslinker [40]. In spite of the relatively low antimicrobial activity of chitosan itself, a small amount of chitosan can significantly improve the antimicrobial effect of PHGH or PHGHE. This complex also effectively improved the wet-strength of cellulose fiber networks or paper.

Moreover, the ionic complex (PEC) with guanidine base polymer E-PHDGC and anionic carboxymethyl cellulose was also developed (CMC) [41]. The morphology of layer-by-layer (LBL) film prepared by the same cationic and anionic combination was revealed via AFM. The LBL film was about 2 nm thick and had granular texture. The E-PHDGC and CMC were then mixed in a slurry of cellulose to prepare an antimicrobial paper incorporated with PEC which showed high antibacterial efficiency within 1 h of contact. Kukharenko et al. [42] prepared a PHGH/bacterial cellulose complex based on physical interaction. The structure of bacterial cellulose is different to plant cellulose, since it forms a much smaller nanostructure.

**Figure 8:**
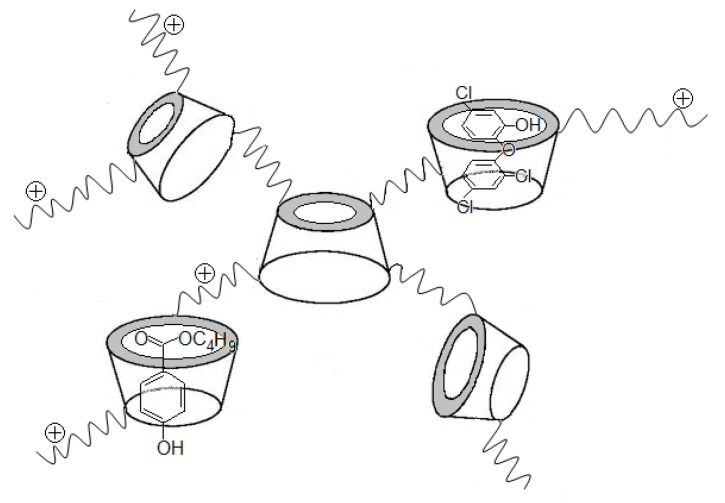
Inclusion of butylparaben and triclosan in water-soluble CPβCD branched polymer. Adapted with permission from ref. [44]. Copyright 2019, John Wiley & Sons.

Cationic cyclodextrin is another effective carrier of antibiotics. The cavity in the molecular ring of cyclodextrin enables it to be a nanocapsule for loading many small molecules. Li et al. [43] linked choline chloride (CC) onto β-cyclodextrin (βCD) through epichlorohydrin (EP) to create cationic β-cyclodextrin polymers (CPβCDs). The molecular weights of the products ranged from 1000 to 8000 Da, depending on the molar ratio of CC, EP, and βCD. The resultant has proper hydrophobic cores for binding guest molecules, antibiotics in particular, thus leading to highly effective antimicrobial agents. The cationic linkages between βCD not only enhanced the water-solubility, but also ensured the high retentions with cellulose fibers for creating antimicrobial hygiene paper products. CPβCDs loaded with butylparaben or triclosan are shown in Figure 8 [40,44]. Butylparaben and triclosan are commercially used broad spectrum non-ionic antibiotics. They need to get into cytoplasm to kill the bacteria by blocking several vital cell functions. The low solubility of these compounds in water limited their use in products. Using CPβCDs as drug carriers significantly improved the water solubility of these antibiotic compounds by 22 times [40].

The complex of CPβCDs and butylparaben or triclosan retained excellent antimicrobial activity, and can be readily incorporated into cellulose fiber networks [45], leading to highly effective antimicrobial paper. It is worth nothing that the antimicrobial mechanism of butylparaben and triclosan is based on the interference of the metabolism function of bacteria, thus raising concerns associated with drug resistance. In contrast, the deactivation mechanism of guanidine-based polymers relies on the physical or electrostatic association between the polymer the cell membrane of bacteria. Such interaction damages or disintegrates the cell membrane, causing the leakage of intracellular components or making massive amounts of cytoplasm leave from bacteria. As a result, the killing of bacteria occurs, meanwhile eliminating the drug-resistant issues. This unique feature also promotes the application of guanidine-based polymers in a variety of areas [46]. In fact, drug resistance has always been an issue in designing antimicrobial agents. Much efforts have been made in the past, including antimicrobial hydrogels which have been developed for tackling such a problem [47]. On the other hand, broad-spectrum antimicrobial activity of polymers and agents is also important, which allows the deactivation of both Gram positive (e.g., *Staphylococcus aureus*) and Gram negative (e.g., *E. coli*) bacteria. Some cationic polymers were specially designed against Gram positive bacterium, as reported by Thoma et al. [48].

## 6. Rendering Biodegradable Polymers and Cellulose-Based Foam Materials Antimicrobial

To test whether incorporation of guanidine-based polymer will retard biodegradation of plastic films, Wang et al. prepared biodegradable antimicrobial film composed of poly(butylene adipate–*co*–terephthalate) (PBAT), thermoplastic starch, and PHGH [49]. PBAT is the number one synthetic biodegradable polymer in terms of world-world productivity at the moment. Incorporation of PHGH does not inhibit, but slows down, biodegradation of PBAT/starch film in soil; 1 wt % of PHGH in film was sufficient to reduce bacteria growth by more than four orders of magnitude. This is the first attempt ever reported on rendering biodegradable polyester antimicrobial using guanidine-based polymer, as well as the impact on biodegradable behavior. As a further extension of the work above, Wang et al. [50] co-extruded PHGH and starch to form antimicrobial thermoplastic starch (APTS), then compounded APTS with linear low-density polyethylene in the presence of a compatibilizer, polyethylene-grafted maleic anhydride. The resulting beads were used to prepare antimicrobial film via a blown filming process. The antimicrobial films were confirmed non-leaching by a ring diffusion test. The films containing more than 12.8 wt % of APTS, i.e., 2% of PHGH, showed more than 4 logs of microbial reduction. Mechanical strengths of APTS containing films were 50% to 60% of pure LLPDE film. 

Wei et al. [51] prepared antimicrobial PBAT films by a series of reactive extrusion. The non-leaching antimicrobial biodegradable PBAT was obtained in the presence of 2,2′-(1,3-phenylene)-bis (2-oxazoline) (PBO) as a free-radical promoter. Finally, the antimicrobial PBAT films were produced using a blown film extrusion system. The resulting film reduced the bacterial content by more than 2 logs with a very fast killing rate. Recently, polylactide (PLA) films including antimicrobial additives were also reported [52]. It is worth noting that PLA is an important and well-received biodegradable polymer apart from PBAT, and mainly prepared via fermentation processes. In addition, Brzezinska et al. [53] utilized polyhexamethylene guanidine derivatives to prepare antimicrobial polycaprolactone (PCL), another type of biodegradable polymer.

Heydarifard et al. [54] (Heydarifard, Pan et al. 2017) incorporated APTS into a cellulose network to create antibacterial cellulosic foam as a green-based filter for water clarification. *E. coli* culture test confirmed that 5% wt APTS endowed the final cellulose foam paper with excellent antimicrobial activity. The ring diffusion test showed that antimicrobial reagent did not leak from the foam matrix to the surroundings, demonstrating the long-term effectiveness and durability of the product. In addition to antimicrobial foam for water disinfection, the guanidinium-functionalized nanofiltration membrane was also reported for both antifouling and antimicrobial applications [55].

## 7. Gemini Antimicrobial Surfactants and Polymers

**Figure 9:**
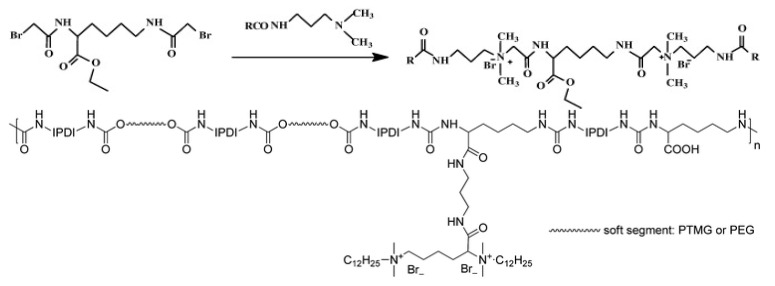
**Upper**: Lysine-based gemini surfactant; **lower**: Schematic structure of waterborne polyurethane (WPU). Adapted with permission from ref. [56]. Copyright 2019, John Wiley & Sons.

Geminis are “bisurfactants”, i.e., surfactants linked together by a spacer (see Figure 9). The spacer can be short or long, rigid or flexible, polar or non-polar [56]. Geminis have much higher surface activity and lower critical micelle concentration (CMC) than comparable traditional surfactants. They usually form large micelles composed of 40 to 80 molecules. The shapes of gemini micelles range from giant wormlike structures to spheres and vesicles, depending on subtle internal and external parameters. The effect of spacer structures on gemini surface properties has been extensively and systematically studied [57,58]. Generally, short hydrophobic spacers favor long non-spherical micelles, while the side of the charge does not affect micelle morphology. Geminis have very strong solubilization ability, i.e., solubilize insoluble compounds in water.

Cationic geminis have attracted lots of attention in antimicrobial research, due to the strong electrostatic binding between a negatively charged cell surface and a positive charge on the surfactant. The much stronger surface activity of a gemini is expected to have a stronger ability to break the cell membrane. Cationic geminis are also incorporated into polymers for two reasons. First, driven by increase of entropy, polyelectrolytes can have stronger bindings to objects that bear multiple opposite charges. Second, polymers can be prepared into non-leaching materials, granting both persistent activity and less contamination to the surroundings. Similar to QAS discussed above, synthesis of polymers containing cationic geminis has two major routes: Appending geminis to polymer backbones or polymerizing monomers that contain gemini.

Tan et al. [59] synthesized a series of l-lysine gemini surfactants with high antimicrobial efficiency. Their work introduced a pendant ester group that can potentially be used to synthesize polymers containing gemini. Based on this work, Zhang et al. [60] synthesized a series of novel l-lysine gemini surfactants containing vinyl groups. These monomers can be conveniently polymerized into polymers and copolymers through free radical polymerization or atom transfer radical polymerization. They synthesized polymers from these monomers and compared the antibacterial and antifungal activities using n-dodecyl-trimethylammonium bromide (DTAB) as reference. The surface activity and antimicrobial activities of their polymers are superior to DTAB. The waterborne polyurethane was also synthesized with gemini quaternary ammonium salt [61]. They dispersed waterborne polyurethane (WPU) in aqueous phase with PSA contained in emulsion. The emulsion was deposited on plate layer-by-layer—up to three layers—in order to improve homogeneous distribution of components. The resultant had GWPU relatively concentrated on the surface. This coating is hydrophilic but does not swell in water. The antibacterial activity of the coating was confirmed by the contact killing test.

**Figure 10:**
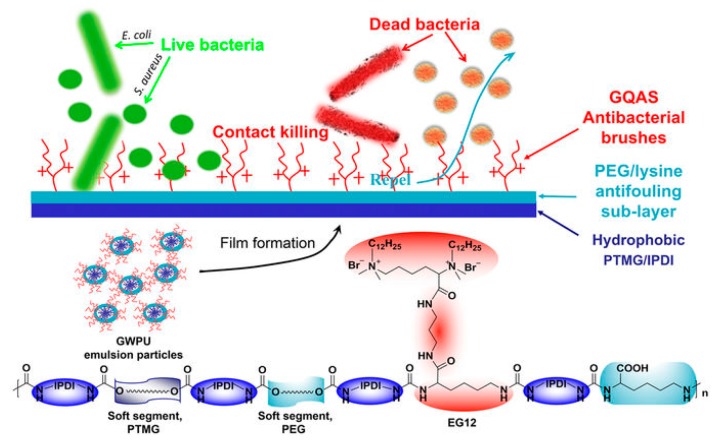
A three-layer structure of antibacterial and antifouling film. The upper-layer contains GQAS that can break cell membrane and kill bacteria. The middle-layer contains steric polymers that can prevent deposition of cell residues on the surface. The base-layer is a hydrophobic substrate depending on different applications. Adapted with permission from ref. [61] Copyright 2019, Springer Nature.

He et al. [62] further developed a novel surface structure consisting of a contact-active antibacterial upper-layer and an antifouling sub-layer derived from antimicrobial polymer [61]. This structure effectively solved the problem of dead cells blocking the antibacterial surface (Figure 10). The antifouling sub-layer is composed of PEG and l-lysine, which can repel proteins and residues from dead cells. The GWPU upper-layer was reported to have high permanent antibacterial activity [61]. The persistent antibacterial activity was confirmed by contact killing test and shake-flask method.

Based on a similar approach, the amphiphilic copolymers containing the antibiotic drug and quaternary ammonium salts also synthesized, leading to a water-soluble polymer with excellent and broad-spectrum antimicrobial activities [63]. Xu et al. [64] synthesized a series of comb-like ammonium polyionenes containing aliphatic side chains. Similar to geminis, the antimicrobial activity of ionene-based polymers depends on the length of spacer between two quaternary ammonium groups and pendent hydrophobic chain length. They found that the (6,7,4)-ionene has much higher antimicrobial activity (as indicated by the MIC) than other samples. The antimold activity of several ionenes was tested by the contact killing test. It was found that the hydrophobic chain length should be long enough for effective antimicrobial activity. The hydrophobic/hydrophilic balance was also found to play an important role on lowering the MIC. Uppu et al. [65] also reported the importance of tunable hydrophobic chains in antimicrobial application, even for enhancing in vivo activity. Overall, the proper balance between the quaternary ammonium groups and the hydrophobic chain (either pendent or backbone) is critical for maximizing the antimicrobial performance. This is also consistent with the mechanisms of pathogen deactivation addressed in the introduction.

To summarize the performance of antimicrobial polymers addressed above, Table 1 provides a comparison of selected antimicrobial polymers and biocides, including some which were not included in the review, in terms of their minimum inhibitory concentrations (MICs) against *E. coli* and/or *S. aureus*. Clearly, star polymer with guanidine arms showed the best performance in terms of MIC against *E. coli*. However, from a practical point of view, guanidine-modified starch containing 15% PHGH (MIC value against *E. coli* is 32.5 μg/mL) is most promising for rendering cellulose fibers or paper packaging, due to its easiness in scaling-up application and cost-effectiveness [21]. On the other hand, chitosan, a well-known antimicrobial polymer, has a relatively high MIC and requires a high dosage to achieve the targeted effect. It is worth noting that even the work cited above has been mainly focused on deactivating *E. coli*, whereas the majority of antimicrobial polymers listed in Table 1 are effective against a broad spectrum of pathogens, i.e., both Gram-negative and Gram-positive bacteria.

## 8. Conclusions

Polymers containing positive charges and hydrophobic chains are promising antimicrobial reagents that have broad antimicrobial spectrum and persistency. The antimicrobial activities of polymers are governed by the balance among solubility, charge density, and hydrophobicity of said polymers. The specific mechanism of bactericidal effect still needs more research to uncover. However, it has been well documented that cationic polymers deactivate the bacteria, mainly via physical interaction or damaging of the membrane of bacterial cells, thus eliminating the concerns associated with drug resistance. Among various cationic antimicrobial polymers, guanidine-based polymers and gemini-type polymers are versatile and powerful, with very high killing efficiency and extremely low MICs. More importantly, guanidine-based polymers could very effectively render polysaccharides, cellulose-based materials, and paper packaging, in particular, antimicrobial, thus extending the antimicrobial application from conventional synthetic materials to green-based materials. Conjugates between antimicrobial polymer and natural abundant polysaccharides, such as cellulose and starch, have the advantage of being lower in cost and more environmentally friendly. Using a proper coupling agent or macromonomer, the guanidine chains could be effectively incorporated into film, paper, and coated products for various applications, including antimold. Non-leaching feature of such polymers enhances the long-term effectiveness. Moreover, the antimicrobial starch was successfully applied in pre-commercial trials, creating novel antimicrobial paper packaging. Overall, cationic polymers tailored with guanidine are very promising in rendering polysaccharides antimicrobial.

## Figures and Tables

**Table 1:** Comparison of antimicrobial activities of antimicrobial polymers in terms of minimum inhibitory concentrations (MICs) and type of bacteria.

Antimicrobial Polymer	MIC (μg/mL)		Reference
	*Escherichia coli*	*Staphylococcus aureus*	
Chitosan	1280		[39]
*Hibiscus surrattensis* L. calyces essential oil	38 ± 10	54 ± 40	[66]
Epichlorohydrin-crosslinked PHGH	7.8		[46]
Copper(II) coordination polymer	250	250	[67]
Amphiphilic Peptide AP3	8.3	4.15	[68]
Star Polymer with guanidine arms	1.56		[31]
PFQ copolymer containing gemini	54	54	[60]
Poly(methacryl guanidine hydrochloride)	113	-	[69]
PEGylated dopamine ester	3120	3120	[70]
Quaternized polycarbonates with propyl and hexyl side chains	31	4	[71]
(6,7,4) ammonium polyionenes (6,7,12) ammonium polyionenes	8 62.5		[64]
Guanidine copolymer-based nanoparticles	8	2	[30]
